# Drug susceptibility surveillance of influenza viruses circulating in the United States in 2011-2012: application of the WHO antiviral working group criteria

**DOI:** 10.1111/irv.12215

**Published:** 2013-12-02

**Authors:** Margaret Okomo-Adhiambo, Ha T Nguyen, Anwar Abd Elal, Katrina Sleeman, Alicia M Fry, Larisa V Gubareva

**Affiliations:** a Influenza Division, National Center for Immunization and Respiratory Diseases, Centers for Disease Control and PreventionAtlanta, GA, USA; b Battelle Memorial InstituteAtlanta, GA, USA

**Keywords:** Influenza, neuraminidase inhibition, oseltamivir, zanamivir

## Abstract

**Background:**

Assessing susceptibility of influenza viruses to neuraminidase (NA) inhibitors (NAIs) is primarily done in NA inhibition (NI) assays, supplemented by NA sequence analysis. However, two factors present challenges for NI assay data interpretation: lack of established IC_50_ values indicative of clinically relevant resistance and insufficient harmonization of NI testing methodologies among surveillance laboratories. In 2012, the WHO working group on influenza antiviral susceptibility (WHO-AVWG) developed criteria to facilitate consistent interpretation and reporting of NI assay data.

**Methods:**

The WHO-AVWG classification criteria were applied in interpreting NI assay data for two FDA-licensed NAIs, oseltamivir and zanamivir, for viruses collected in the United States during the 2011–2012 winter season.

**Results:**

All A (H1N1)pdm09 viruses (*n* = 449) exhibited normal inhibition by oseltamivir and zanamivir, with the exception of eight viruses (1·8%) with highly reduced inhibition by oseltamivir, which carried the H275Y marker of oseltamivir resistance. A (H3N2) viruses (*n* = 978) exhibited normal inhibition by both NAIs, except for one virus with highly reduced inhibition by zanamivir due to the cell culture-selected NA change, Q136K. Type B viruses (*n* = 343) exhibited normal inhibition by both drugs, except for an isolate with reduced inhibition by both NAIs that had the cell culture-selected A200T substitution.

**Conclusions:**

WHO-AVWG classification criteria allowed the detection of viruses carrying the established oseltamivir resistance marker, as well as viruses whose susceptibility was altered during propagation. These criteria were consistent with statistical-based criteria for detecting outliers and will be useful in harmonizing NI assay data among surveillance laboratories worldwide and in establishing laboratory correlates of clinically relevant resistance.

## Introduction

Monitoring influenza antiviral susceptibility has become a vital part of virological surveillance within the WHO Global Influenza Surveillance and Response System (WHO-GISRS). The information gained is essential for making decisions with regard to drug-use recommendations, clinical care, outbreak management, and pandemic preparedness. Neuraminidase (NA) inhibitors (NAIs) are currently the only class of antiviral drugs effective against influenza infections.^1^ Orally administered oseltamivir and inhaled zanamivir are FDA-approved, while newer NAIs include intravenously administered peramivir,^2^ which is licensed in Japan, South Korea, and China, and the long-acting inhaled laninamivir (CS-8958), licensed in Japan.^3^

The unexpected emergence and global spread of oseltamivir-resistant A (H1N1) viruses carrying the H275Y mutation in the NA during 2007–2009 4–7 reinforced the importance of drug susceptibility surveillance. The oseltamivir-resistant A (H1N1) viruses were displaced by the A (H1N1) pdm09 viruses that emerged in April 2009.^8^ Resistance to oseltamivir has remained low among A (H1N1) pdm09 viruses circulating in the United States 9,10 and other countries.^11^–^13^ However, a worrisome trend was noticed when the majority of detected H275Y viruses were collected from patients with no known exposure to oseltamivir.^9^–^11^ Moreover, in 2011, a cluster of cases with oseltamivir resistance was detected in Australia.^14^,^15^ Such potential for emergence and spread of NAI-resistant viruses and the limited therapeutic options available highlight the need for sustained NAI susceptibility surveillance among globally circulating influenza viruses.

For surveillance purposes, susceptibility to NAIs is assessed in either the fluorescent 16 or the chemiluminescent NA inhibition assay.^17^ However, the fifty percent inhibitory concentration (IC_50_) values generated in the NI assay are affected by variations in assay protocols,^18^–^21^ making it difficult to compare IC_50_ data generated in different laboratories.^22^ Moreover, there is no established cutoff IC_50_ value which discriminates between viruses susceptible to NAIs and viruses with clinically relevant resistance. The lack of standardization in NI assay methodologies and the resulting IC_50_ variability create challenges in sharing and interpreting IC_50_ data among surveillance laboratories within the WHO-GISRS.

In June 2011, the WHO working group on surveillance of influenza antiviral susceptibility (WHO-AVWG) was created with the mandate to develop practical approaches for antiviral susceptibility surveillance, provide advice on appropriate surveillance strategy, and guide the interpretation of laboratory surveillance data.^22^ In an effort to harmonize and ensure consistency in reporting and data analysis, the WHO-AVWG agreed on criteria to define influenza viruses as exhibiting normal, reduced, or highly reduced NA inhibition, based on the fold change of their IC_50_ compared to reference IC_50_ values.^22^ Application of these criteria does not negate the need for NA sequence analysis, because viruses displaying reduced or highly reduced inhibition must be sequenced to identify any underlying amino acid residue changes in the NA. Subsequently, highly reduced inhibition in the NI assay coupled with the identification of an established marker of clinically relevant resistance (e.g., H275Y substitution) is interpreted as resistance, while in other instances the interpretation remains uncertain. Although it is unknown what reduced inhibition means clinically, it is important to monitor such viruses for public health purposes.

The WHO-AVWG criteria list ranges of IC_50_ fold change specific to type A and type B viruses; however, they do not specify which reference IC_50_ should be used to calculate the fold change and thus remain ambiguous in this respect. In this study, we applied the WHO-AVWG criteria in interpreting the NAI susceptibility of influenza A and B viruses that circulated in the United States during the 2011–2012 winter influenza season. We examined different options for determining fold changes in IC_50_ of test viruses. All three approaches effectively enabled the detection of viruses carrying known markers of NAI resistance (e.g., H275Y), as well as viruses that acquired cell culture-selected changes (e.g., Q136K), and were consistent with the statistical-based criteria previously applied for detecting outliers.

## Materials and methods

### Viruses

Seasonal influenza A and B viruses collected in the United States between October 01, 2011, and September 30, 2012, were submitted to the WHO Collaborating Center for Surveillance, Epidemiology and Control of Influenza at the CDC in Atlanta, Georgia, the United States. Variant influenza A (H3N2)v viruses collected in the United States during the 2011–2012 season were also included in the study. All viruses were propagated in MDCK cells (ATCC, Manassas, VA, USA) or eggs and antigenically characterized by the hemagglutination inhibition (HI) prior to antiviral susceptibility testing.

### Neuraminidase inhibitors

Oseltamivir (carboxylate), the active compound of the ethyl ester prodrug oseltamivir phosphate, was supplied by Hoffmann-La Roche (Basel, Switzerland) and zanamivir by GlaxoSmithKline (Uxbridge, UK).

### Neuraminidase inhibition assay

Susceptibility of virus isolates to oseltamivir and zanamivir was assessed in the fluorescent NI assay, using the NA-Fluor™ Influenza Neuraminidase Assay Kit (Applied Biosystems, Foster City, CA, USA) with modifications to the manufacturer's protocol.^23^

### Data analysis

Raw fluorescent NI assay data (expressed as RFU) were plotted against drug concentration (nm) to determine IC_50_ values, using Jaspr v1.2 curve-fitting software (CDC, Atlanta, GA, USA).^23^ Box-and-whisker plots used to identify extreme IC_50_s (outliers) and to determine baseline IC_50_s were generated using sas 9.3 software (SAS Institute, Cary, NC, USA), as previously described.^24^

### Interpretation of IC_50_ values for WHO-AVWG criteria

Fold changes in IC_50_ were determined by different approaches where IC_50_s of test viruses were divided with (i) the IC_50_ of an influenza type-specific, drug-susceptible reference virus tested in the same assay as the test virus (ii) the median IC_50_ among type-specific, drug-susceptible reference viruses obtained from different assays, and (iii) the median IC_50_ among test viruses or mean IC_50_ (outliers excluded) by respective drug and influenza type and subtype. The fold changes in IC_50_ were interpreted based on the WHO-AVWG criteria established for influenza A and B viruses.^22^ Influenza A viruses with <10-fold change in IC_50_ were characterized as exhibiting normal inhibition by the respective NAI, while those with 10- to 100-fold and >100-fold change as exhibiting reduced and highly reduced inhibition, respectively. The same criteria were applied to influenza B viruses, but using <5-fold, 5- to 50-fold, and >50-fold changes in IC_50_ to characterize viruses as exhibiting normal, reduced, and highly reduced inhibition, respectively. We compared the outcome of each approach to the results from the statistical-based method for the detection of outliers.^24^

### Sequence analysis

Viruses showing reduced and highly reduced inhibition were assessed by genetic analysis, using pyrosequencing 25 and/or Sanger sequencing,^26^ to detect known and/or novel NA markers associated with reduced susceptibility to NAIs. Amino acid substitutions in the NA are listed according to straight numbering throughout the text.

## Results

The first approach to determine fold change in IC_50_ values compared IC_50_s of test viruses to those of influenza type-specific NAI-susceptible reference viruses, tested in the same NI assay run (Table 1). Compared to the A/California/07/2009 (H1N1)pdm09 reference virus, all A (H1N1) pdm09 viruses (*n* = 449) exhibited normal inhibition by oseltamivir and zanamivir, with exception of eight isolates exhibiting highly reduced inhibition by oseltamivir. NA sequence analysis of these eight viruses revealed the H275Y oseltamivir resistance conferring substitution. Pyrosequencing and single-nucleotide polymorphism (SNP) analysis revealed that all eight viruses comprised 100% H275Y viral populations, with exception of one virus, A/Delaware/03/2012, which was a mix of 40% wild-type virus (H275) and 60% mutant (H275Y). All A (H3N2) viruses (*n* = 978) exhibited normal inhibition by oseltamivir and zanamivir (Table 1), with exception of A/New York/02/2012, which exhibited highly reduced inhibition by zanamivir, and had a Q136Q/K mix in the NA comprising 44% wild-type virus (Q136) and 56% mutant (Q136K). The Q136K substitution was not detected in matching original clinical specimen and is therefore considered a cell culture artifact.

**Table 1:** NA inhibition of influenza A and B viruses based on fold change in IC_50_ of test viruses assessed in the NA-Fluor™ NI assay

Type	Subtype	NA inhibitors	NA inhibition*	Fold change in IC_50_ of test viruses (No. of viruses):			Amino acid changes in the NA
				Approach #1**	Approach #2†	Approach #3††	
Influenza A (*n* = 1583)	H1N1pdm09 (*n* = 449)	Oseltamivir	Normal	0–6 (441)	0–6 (441)	1–7 (441)	–
			Reduced	–	–	–	–
			Highly reduced	319–1474 (8)	182–1403 (8)	213–1637 (8)	H275Y
		Zanamivir	Normal	0–6 (449)	1–6 (449)	1–6 (449)	–
			Reduced	–	–	–	–
			Highly reduced	–	–	–	–
	H3N2 (*n* = 978)	Oseltamivir	Normal	0–4 (978)	0–4 (978)	0–7 (978)	–
			Reduced	–	–	–	–
			Highly reduced	–	–	–	–
		Zanamivir	Normal	1–6 (977)	1–6 (977)	0–5 (977)	
			Reduced	–	–	91 (1)	
			Highly reduced	132 (1)	132 (1)	–	Q136Q/K
	H3N2v (*n* = 156)	Oseltamivir	Normal	0–2 (155)	0–1 (155)	0–1 (155)	–
			Reduced	29 (1)	25 (1)	35 (1)	S245N + S247P
			Highly reduced	–	–	–	–
		Zanamivir	Normal	2–5 (155)	2–4 (155)	0–1 (155)	–
			Reduced	–	–	70 (1)	S245N + S247P
			Highly reduced	223 (1)	199 (1)	–	S245N + S247N
Influenza B (*n* = 343†††)	–	Oseltamivir	Normal	1–2 (112)	0–3 (341)	0–4 (342)	–
			Reduced	–	5–8 (2)	6 (1)	A200A/T; G70R + T72A
			Highly reduced	–	–	–	–
		Zanamivir	Normal	1–2 (112)	1–3 (342)	0–2 (342)	–
			Reduced	–	7 (1)	5 (1)	A200A/T
			Highly reduced	–	–	–	–

* Influenza A viruses – normal inhibition: <10-fold change; reduced inhibition: 10- to 100-fold change; highly reduced inhibition: >100-fold change. Influenza B viruses – normal inhibition: <5-fold change; reduced inhibition: 5- to 50-fold change; highly reduced inhibition: >50-fold change.

** Fold changes determined by dividing IC_50_s of test viruses by IC_50_s of NAI-susceptible type-specific reference viruses tested in same assay. Reference viruses – A/California/07/2009 (H1N1)pdm09 H275 wild-type and B/Rochester/02/2001 D198 wild-type viruses.

† Fold changes determined by dividing IC_50_s of test viruses by median IC_50_s of type-specific reference viruses from various assays (70 assays for A/California/07/2009 and 11 assays for B/Rochester/02/2001).

†† Fold changes determined by dividing IC_50_s of test viruses by median IC_50_s for virus type/subtype.

††† Includes 112 isolates tested in assays where influenza B reference viruses were included, and 231 isolates tested in assays without influenza B reference viruses.

All influenza B viruses (*n* = 112) tested in the same assay run as B/Rochester/02/2001 reference virus exhibited normal inhibition by oseltamivir and zanamivir in the first approach for determining IC_50_ fold change (Table 1). Of note, only 112 of the 343 influenza B isolates analyzed in this study were tested in assays incorporating the type B reference virus. The remaining isolates (*n* = 231) were tested in assays incorporating only the type A reference virus, which was standard practice at the CDC prior to the publication of the WHO-AVWG criteria. The CDC's algorithm for antiviral testing has since been revised to incorporate both type A and B reference viruses whenever both virus types are tested in the same assay.

In the second approach to determine IC_50_ fold change, IC_50_s of test viruses were divided by a common reference IC_50_ value – the median IC_50_ of influenza type-specific reference viruses, derived from different NI assays (Table 1). The NA inhibition profiles for influenza A viruses were similar to those obtained using the previous approach. However, for influenza B viruses (*n* = 393), the isolate B/Alabama/03/2012, earlier characterized as showing normal inhibition by oseltamivir, exhibited reduced inhibition by the drug in the second approach. This isolate possessed the substitutions, G70R and T72A that are located at the stalk region of the NA, and therefore not expected to influence NA enzyme inhibition. Another isolate, B/California/03/2012, not among viruses analyzed by the first approach, exhibited reduced inhibition by oseltamivir and zanamivir by the second approach. This isolate possessed an A200A/T mix in the NA, comprising 69% wild-type (A200) and 31% mutant (A200T) viruses. The matching clinical specimen comprised 95% and 5% wild-type and mutant viruses, respectively, indicating that the A200T substitution provided some growth advantage in MDCK cells.

The third approach to determine IC_50_ fold change which compared IC_50_s of test viruses with the median IC_50_ determined for the entire set of viruses of the same type and subtype (Table 1) yielded results similar to those of the previous approaches. However, A/New York/02/2012 with the Q136Q/K mix, previously characterized by the first and second approaches as showing highly reduced inhibition by zanamivir, exhibited only reduced inhibition. The isolate, B/Alabama/03/2012, with G70R and T72A substitutions, which exhibited normal and reduced inhibition by oseltamivir in the first two approaches, respectively, demonstrated normal inhibition by the drug in the third approach.

Variant influenza A (H3N2)v viruses collected from an outbreak in humans during the study period were also analyzed (Table 1). All (H3N2)v isolates (*n* = 156) were interpreted as showing normal inhibition by oseltamivir and zanamivir compared to the respective median IC_50_s for A/California/07/2009 (H1N1)pdm09 reference virus. The exception was A/Ohio/88/2012, which demonstrated reduced inhibition by oseltamivir and highly reduced inhibition by zanamivir and possessed the NA changes, S245N and S247P. However, compared to respective median IC_50_s for oseltamivir and zanamivir among A (H3N2)v viruses, A/Ohio/88/2012 exhibited reduced inhibition by both drugs.

The results of the standard statistical method used at the CDC to determine baseline IC_50_s and detect outliers for oseltamivir and zanamivir, for each virus type/subtype (Table 2), were consistent with IC_50_ data interpretations based on the WHO-AVWG criteria (Table 3). All extreme outliers for oseltamivir (*n* = 8) among A (H1N1) pdm09 viruses had the H275Y substitution and exhibited highly reduced inhibition by the drug. However, all A (H1N1)pdm09 viruses that were mild outliers for oseltamivir (*n* = 4) and zanamivir (*n* = 2) exhibited normal inhibition by the respective drugs. There were no extreme outliers for oseltamivir among A (H3N2) viruses, but the only extreme outlier for zanamivir among this subtype, A/New York/02/2012 with Q136Q/K mix in the NA, exhibited highly reduced inhibition by the drug. All mild outliers for oseltamivir (*n* = 27) and zanamivir (*n* = 30) among A (H3N2) viruses exhibited normal inhibition by both drugs. There were no extreme outliers for oseltamivir or zanamivir among B viruses, but the detected mild outliers for oseltamivir (*n* = 5) exhibited normal inhibition by the drug. The exception was one virus, B/California/03/2012 with A200A/T mix in the NA, which also a mild outlier for zanamivir and exhibited reduced inhibition by both NAIs.

**Table 2:** Statistical analyses of neuraminidase inhibitor susceptibility data of influenza viruses, assessed in the NA-Fluor™ NI assay

Influenza type and subtype	NAI	IC_50_, nm										
		All isolates (including outliers)							Isolates (excluding outliers)			
		No. of isolates (*n*)*	Min–Max**	Q1***	Median†	Q3††	IQR†††	Statistical cutoff‡	No. of isolates (*n*)‡‡	Min–Max‡‡‡	Mean (±SD)§	Median§§
A (H1N1) pdm09	Oseltamivir	449	0·10–294·72	0·15	0·18	0·22	0·07	0·43	437	0·10–0·41	0·19 ± 0·05	0·18
	Zanamivir	449	0·10–1·03	0·15	0·17	0·19	0·04	0·31	447	0·10–0·31	0·17 ± 0·03	0·17
A (H3N2)	Oseltamivir	978	0·03–0·76	0·10	0·11	0·13	0·03	0·22	951	0·03–0·22	0·11 ± 0·03	0·11
	Zanamivir	978	0·12–23·75	0·22	0·26	0·30	0·08	0·54	947	0·12–0·54	0·26 ± 0·07	0·25
A (H3N2)v	Oseltamivir	156	0·08–5·30	0·13	0·15	0·17	0·04	0·29	155	0·08–0·21	0·15 ± 0·03	0·15
	Zanamivir	156	0·27–35·74	0·46	0·51	0·60	0·14	1·02	155	0·27–0·74	0·52 ± 0·09	0·51
B	Oseltamivir	343	0·83–53·02	6·49	8·54	10·39	3·90	22·09	338	0·83–21·73	8·77 ± 3·13	8·51
	Zanamivir	343	0·40–5·11	0·93	1·11	1·32	0·39	2·49	342	0·40–2·38	1·14 ± 0·32	1·11

* Tested isolates, including outliers.

** Minimum to maximum IC_50_ values, all viruses.

*** Q1: first quartile (25th percentile), all viruses.

† Median (Q2): second quartile (50th percentile), all viruses.

†† Q3: Third quartile (75th percentile; X_0·75_), all viruses.

††† IQR: Interquartile range (IQR=Q3-Q1).

‡ Statistical IC_50_ cutoff for NAI susceptibility, set at 3 interquartile ranges (3IQR) from the 75th percentile (=X_0·75_ + 3IQR). Outliers with IC_50_ above cutoff and >10-fold mean IC_50_ of drug were characterized as extreme outliers. Mild outliers were isolates with IC_50_ >X_0·75_ + 3IQR, but >2-fold <10-fold that of the mean IC_50_ of the drug.

‡‡ Number of isolates analyzed to determine mean and median drug IC_50_s, outliers excluded.

‡‡‡ Minimum to maximum IC_50_ values, outliers excluded.

§ Mean and standard deviation (SD) of IC_50_ values, outliers excluded.

§§ Median of IC_50_ values, outliers excluded.

**Table 3:** Statistical detection of outliers compared to WHO-AVWG criteria

Virus type/subtype	NA inhibitor	Outlier type	No. of outliers (*n*)	IC_50_, nm (fold change)*	NA inhibition**	Amino acid changes in the NA (No. of outliers)***
A (H1N1)pdm09 (*n* = 449)	Oseltamivir	Extreme	8	38-27-294·72 (213–1637)	Highly reduced	H275Y (8)
		Mild	4	0·49–1·22 (3–6)	Normal	S110F (1); T135N + D292N (1)
	Zanamivir	Mild	2	0·36–1·03 (2–6)	Normal	S110F (1)
A (H3N2) (*n* = 978)	Oseltamivir	Mild	27	0·23–0·76 (2–7)	Normal	I222T + D151D/N (1)
	Zanamivir	Extreme	1	23·75 (91)	Reduced	Q136Q/K (1)
		Mild	30	0·55–1·28 (2–5)	Normal	D151D/N (1); T148I + D251V (1); T148T/I (1); D151D/N (1); D151D/G + D251V (1)
A (H3N2)v (*n* = 156)	Oseltamivir	Extreme	1	5·30 (35)	Reduced	S245N + S247P (1)
	Zanamivir	Extreme	1	35·74 (70)	Reduced	S245N + S247P (1)
B (*n* = 343)	Oseltamivir	Mild	1	53·02 (6)	Reduced	A200A/T (1)
		Mild	4	23·50–32·27 (3–4)	Normal	G70R + T72A (2); K343E (1); K107N + K343E (1)
	Zanamivir	Mild	1	5·11 (5)	Reduced	A200A/T (1)

* Compared to median IC_50_ for drug by virus type/subtype.

** Based on fold change determined by comparing IC_50_ of test viruses to the median IC_50_ by virus type/subtype.

*** Number of outliers with amino acid changes in the NA, based on available sequence information.

## Discussion

Previously, the Global Neuraminidase Inhibitor Susceptibility Network (NISN), now known as the isirv Antiviral Group (isirv-AVG), set criteria for defining NAI resistance as either IC_50_ >3 standard deviations (SD) from the mean (or median) or IC_50_ >10-fold mean (or median) for the influenza type and subtype, and NAI.^27^ Various surveillance studies also set statistical criteria for the detection of outliers and interpretation of NAI susceptibility.^20^,^24^,^26^,^28^–^30^ In this study, the WHO-AVWG criteria were consistent with statistical methods we previously used 24 for detecting outliers among the influenza A and B viruses. The criteria enabled the detection of viruses carrying a well-characterized marker of oseltamivir resistance, H275Y, among eight influenza A (H1N1)pdm09 viruses, as well as viruses with cell culture-selected changes, such as Q136K, in one A (H3N2) virus.

The WHO-AVWG criteria are beneficial for laboratory surveillance, because they facilitate sharing of phenotypic NAI susceptibility data globally in a simplified and overall reproducible way. However, translating the WHO-AVWG criteria from laboratory to national surveillance is not straightforward. At present, only those viruses that exhibit highly reduced inhibition in the NI assay and contain commonly detected molecular markers associated with laboratory resistance are reported as resistant in the CDC FluView weekly report on U.S. national influenza virological surveillance.^31^ Specifically, the substitutions H275Y in viruses of A (H1N1) and A (H1N1)pdm09 subtypes as well as E119V and R292K in A (H3N2) viruses are interpreted as molecular markers of oseltamivir resistance because such changes are repeatedly detected in viruses isolated from immunocompetent patients treated with oseltamivir.^32^ As more information is gained, viruses with other markers could be reported as resistant to oseltamivir and other NAIs. The WHO-AVWG criteria remain vague in that they do not specify the exact references to which test viruses should be compared to determine fold change in IC_50_. When a novel virus emerges, there is a need to assess and report its susceptibility to available antiviral medications. For atypical non-seasonal influenza A viruses, such as the A (H3N2)v that caused outbreaks of human illness in the United States in 2011–2012, there are no defined reference viruses susceptible to NAIs, against which their IC_50_s can be compared. The WHO-AVWG criteria do not address such situations. The A (H3N2)v virus with S245N and S247P substitutions, A/Ohio/88/2012, exhibited highly reduced inhibition by zanamivir when its IC_50_ for the drug was compared with that of the type A reference virus, but demonstrated reduced inhibition by the drug, when its IC_50_ was compared to the median IC_50_ for the A (H3N2)v subtype. Although desirable, using subtype-specific reference viruses, as opposed to type-specific viruses, may not be the optimal option because it increases the cost of testing and in certain instances only virus type, but not subtype, may be known at the time of testing. Determining baseline IC_50_s for atypical non-seasonal viruses may facilitate the assessment of NA inhibition against a mean/median IC_50_ for the virus subtype, but baseline IC_50_s may be difficult to accurately define for viruses that are rare in circulation due to small sample sizes.

Comparing test virus IC_50_s to those of an influenza type-specific reference virus provides a streamlined option when reporting NI data to the GISRS.^33^ However, laboratories need to be aware that assay-to-assay fluctuation of IC_50_s for reference viruses may affect fold changes in IC_50_s for test viruses. However, using IC_50_s of reference viruses generated assay-by-assay is a good quality control measure that provides preliminary NA inhibition results and facilitates immediate detection of viruses that may need retesting in the NI assay, or further testing by genetic analysis. When analyzing a batch of viruses tested on different test dates, for example at the end of the surveillance period, it may be practical and prudent to use a common IC_50_ value for the NAI-susceptible reference viruses, such as the median IC_50_ or the mean (minus outliers) to determine fold changes in IC_50_.

Of note, several A (H3N2) viruses characterized by the WHO-AVWG criteria as exhibiting normal inhibition by oseltamivir and zanamivir were detected as mild outliers for the respective NAIs based on the statistical-based method.^24^ These viruses had borderline IC_50_ fold changes just below 10-fold, the cutoff for normal inhibition. The available NA sequences for the mild outliers among A (H3N2) viruses revealed the presence of cell culture-selected changes at residue D151, which have been shown to increase IC_50_s in influenza A viruses.^20^,^26^,^34^ Nevertheless, by identifying outliers with NA changes, the statistical analysis provided additional insights, which may be relevant in certain instances. Therefore, it seems reasonable for the WHO Collaborating Centers that conduct high-throughput antiviral testing to continue performing statistical analyses in addition to applying the WHO-AVWG criteria. If experimental evidence supporting the significance of the NA changes detected in the mild outliers could be obtained, such changes would be added to the list of potential molecular markers of antiviral resistance,^35^ enabling the wider surveillance community to access this information, and include such markers in their monitoring algorithm.

Although the WHO-AVWG criteria are expected to harmonize interpretation and reporting of IC_50_ data, there still remains a lack of consensus on the reference for determining IC_50_ fold changes in test viruses. Moreover, a clinically relevant IC_50_ cutoff value that would discriminate between clinically relevant NAI-susceptible and resistant viruses, regardless of the virus type/subtype or drug, is yet to be determined. Nevertheless, application of the WHO-AVWG criteria, coupled with NA sequence analysis of viruses characterized as having reduced and highly reduced inhibition by NAIs, provides a reliable approach to interpreting and reporting NI assay data across surveillance laboratories globally.

## Conclusion

The application of the WHO-AVWG criteria to the NI assay data of U.S. viruses circulating during the 2011–2012 winter season was successful. The criteria provide a good framework for interpreting IC_50_ data; however, there is need for more evidence to support the interpretations and for further refinement. Continuous review and evaluation of the WHO-AVWG recommendations on NI methodology and testing algorithms will be beneficial to ensure that the criteria remain relevant and appropriate to circulating influenza viruses as more information becomes available.
